# Thermal Fatigue Failure of Micro-Solder Joints in Electronic Packaging Devices: A Review

**DOI:** 10.3390/ma17102365

**Published:** 2024-05-15

**Authors:** Lei Li, Xinyu Du, Jibing Chen, Yiping Wu

**Affiliations:** 1 School of Mechanical Engineering, Wuhan Polytechnic University, Wuhan 430023, China; 2 School of Material Science and Engineering, Huazhong University of Science & Technology, Wuhan 430074, China

**Keywords:** micro-solder joint, thermal fatigue, microstructure, lifetime, electronic packaging

## Abstract

In electronic packaging products in the service process, the solder joints experience thermal fatigue due to temperature cycles, which have a significant influence on the performance of electronic products and the reliability of solder joints. In this paper, the thermal fatigue failure mechanism of solder joints in microelectronic packages, the microstructure changes of the thermal fatigue process, the influence factors on the joint fatigue life, and the simulation analysis and forecasting of thermal fatigue life are reviewed. The results show that the solder joints are heterogeneously coarsened, and this leads to fatigue cracks occurring under the elevated high-temperature phase of alternating temperature cycles. However, the thickness of the solder and the hold time in the high-temperature phase do not significantly influence the thermal fatigue. The coarsened region and the IMC layer thicken with the number of cycles, and the cracks initiate and propagate along the interface between the intermetallic compound (IMC) layer and coarsened region, eventually leading to solder joint failure. For lead-containing and lead-free solders, the lead-containing solder shows a faster fatigue crack growth rate and propagates by transgranular mode. Temperature and frequency affect the thermal fatigue life of solder joints to different degrees, and the fatigue lifetime of solder joints can be predicted through a variety of methods and simulated crack trajectories, but also through the use of a unified constitutive model and finite element analysis for prediction.

## 1. Introduction

At present, in the field of electronic materials, the solder plays a key role in the assembly and connection of chips, as a connection material, providing electrical, thermal, and mechanical continuity in electronic components. The performance and quality of the solder are critical to the integrity of a solder joint, as well as to the overall function of the component. The solder, as a chip connection material, is used in different levels of the continuous manufacturing of electronic components, and acts as an electrical and mechanical connection between silicon chips and pads, in addition to acting as a means of dissipation of the heat generated by semiconductors [1]. In the process of electronic package welding and service, the reliability of electronic equipment is often based on the reliability of solder joints [2,3]. As the size of solder joints gradually decreases, solder joints become the weakest connection link, and 70% of the failures of electronic devices are caused by the failures of packaging and assembly. In the failures of electronic packaging and assemblies, the failure causes of solder joints are mechanical, thermal, electrical, radiation, and chemical. Thermal failure is one of the main reasons for failure [4].

**Figure 1:**
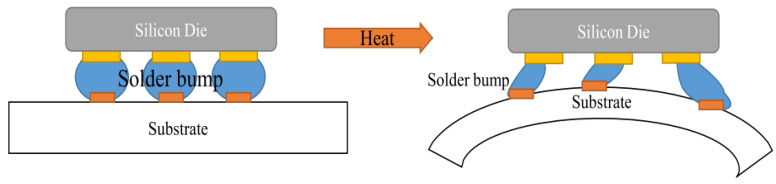
Deformation of solder bump subjected to stress in consideration of CTE mismatch [5].

With the rapid development of microelectronics packaging technology, to improve the integration and reduce the size of devices, the surface mount technology (SMT) assembly, new chip scale packaging (CSP), ball grid array (BGA), and multi-chip module (MCM) packaging technologies are widely used to directly realize the electrical and mechanical connection between different materials through solder joint interconnection, which will inevitably produce stress concentration and dramatically increase its strain level [5]. The coefficient of thermal expansion (CTE) of different materials between layers is different, which will inevitably lead to thermal failure, as shown in Figure 1. The production process and process of composite microelectronics packaging structure are very complicated, and the intermediate process needs to undergo multiple heating and cooling processes, which will inevitably cause residual thermal stress or thermal drop strain [6]. Moreover, a large number of material interfaces that are prone to defects are introduced between layers. As a result, the microelectronic structure itself has a large number of microscopic defects, which will lead to the initiation and expansion of thermal fatigue cracks and eventually failure after the repeated heating and cooling in the packaging process and the continuous heating and cooling caused by working and stopping during use [7]. Due to the high Sn content and high welding temperature in the commonly used lead-free solder joints, the dissolution rate of Cu in the solder joints and the growth rate of intermetallic compounds at the interface are much higher than those in the SnPb solder joints. In addition, as the assembly density is getting higher and higher and the solder joint in the microelectronic device is getting smaller and smaller, the proportion of intermetallic compounds in the entire solder joint is getting larger and larger, and its performance is an important factor affecting the reliability of the entire solder joint.

Power electronic devices, such as gate turn-off thyristors (GTO), high-power transistors (GTR), power MOSFETs, insulated gate bipolar transistors (IGBTs), and integrated gate commutator thyristors (IGCT), are the core components for motor control and power conversion to achieve high temperature, reliability, and long life. All of them are directly packaged and connected between electronic devices by interconnecting solder joints [8,9]. The improvement of power density and conversion efficiency of power electronic devices, as well as the enhancement of safe service reliability, is a worldwide problem that needs urgent breakthroughs [10]. In particular, the IGBT chip is the “heart” of the power electronics industry, which can control and provide high-power power equipment power conversion, effectively improving the energy utilization efficiency, automation, and intelligence level of the equipment. IGBT devices, modules, components, and system devices composed of IGBT chips are widely used in household appliances such as air conditioners and washing machines, as well as high-end industries such as rail transit, smart grid, aerospace, marine drive, new energy, and electric vehicles, especially in strategic industries involving national economic security and national defense security. It should have a service life of at least 30 years. It can be seen that the IGBT module needs to withstand 10^6^–10^7^ power cycles during service, which requires that the IGBT module still has high reliability under long-term service conditions [11].

For high-power electronic devices with large pad structures, factors such as temperature, vibration, and humidity are the main reasons for their failure, among which temperature accounts for the main part of the total failure of power devices [12]. This is because, as it withstands the action of high frequency, high voltage, and high current, the solder joint temperature of the sandwich structure alternates between high and low, and the solder joint experiences a rapid temperature cycle, which makes the brittle intermetallic compound (IMC) between the solder joint interface grow rapidly. In addition, bubbles are easy to include in the packaging and welding process, and with the alternating change of temperature and stress and the mismatch of the coefficient of thermal expansion (CTE), cracks are generated in the tip/front end and the final failure is mainly caused by the failure caused by the alternating temperature load [13]. The reliability of solder joints under thermal cycle conditions can be achieved through reliability tests and analyses, which aim to evaluate and predict the reliability level of integrated circuit electronic devices and provide a reliable reference for the reliability design of the whole machine [14].

In terms of fatigue resistance, some packaging process innovations have been focused on the strain elimination of solder joints. Since the solder joints in ball welded joints provide the connection between the substrate and the packaging both electrically and mechanically, they are susceptible to temperature and mechanical loads. They have broad prospects for future applications such as new-energy vehicles, high-powered devices, and artificial intelligence. The SnxAgxCu (SAC) alloy is the broadest used alternative to lead–tin solder at present and will be for some time to come. To improve the fatigue life of Pd-free solder, some additives can be used to improve their fatigue resistance properties, such as the shear stress, tensile stress of the packaging, the hardness of the solder joint, and the creep resistance. Table 1 lists some of the available innovations of SAC solders in terms of additives to improve the shear stress, creep resistance, tensile stress, and hardness.

Another important part of the investigation on the performance of solder joints is the failure mechanism during service. This research is mainly carried out from the angle of the internal crack of the solder joint and its expansion form. Zhao J. et al. [26] studied the crack growth forms of the solder joints. Most of the cracks in the solder joints are generated and expanded in a transgranular manner under the action of cyclic stress, so that irregular eutectic Sn and acicular Ag_3_Sn intermetallic compounds are formed in the solder joints, among which Ag_3_Sn appears as a strengthening phase, thus hindering the growth of transgranular cracks. As a result, the fatigue crack will spread along the IMC interface of the solder joint in the form of a transgranular/intergranular mixture until it passes through the entire interface of the joint, reducing its performance.

Therefore, taking the thermal fatigue effect between solder and substrate as an example, this paper mainly introduces the current research status of the thermal fatigue of solder joints, the microstructure of intermetallic compounds, the simulation analysis of solder joint reliability, and the research progress of fatigue life prediction of solder joints, to provide a certain reference value for improving the thermal fatigue performance of solder joints.

## 2. Thermal Fatigue Failure Mechanism of Solder Joints

To observe the different morphologies of lead-free solder and lead-containing solder under thermal cycling conditions, higher requirements are put forward for metallographic sampling of solder joints. The general procedure is to insert the solder joint, then release the mold, grind the sample along the longitudinal section of the solder joint, and then, in time, cool the grinding sample with running water. The polishing first uses an Al_2_O_3_ suspension, then uses polishing paste, and finally uses 4%HNO_3_ in the corrosion liquid, and the corrosion time is controlled within 10–20 s to obtain an ideal contrast of the solder matrix. The changes in solder microstructure were observed by scanning electron microscopy (SEM). EDAX (energy dispersive X-ray microanalysis) and XRD (X-ray dispersive) can also be used to identify the components of the solder that produce IMC under thermal cycling.

**Figure 2:**
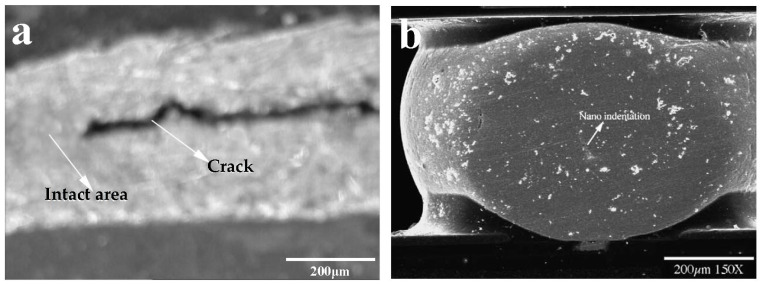
(**a**) Crack in solder joint under fatigue shear load. (**b**) Solder joint in BGA module after 1500 thermal cycles [28].

**Figure 3:**
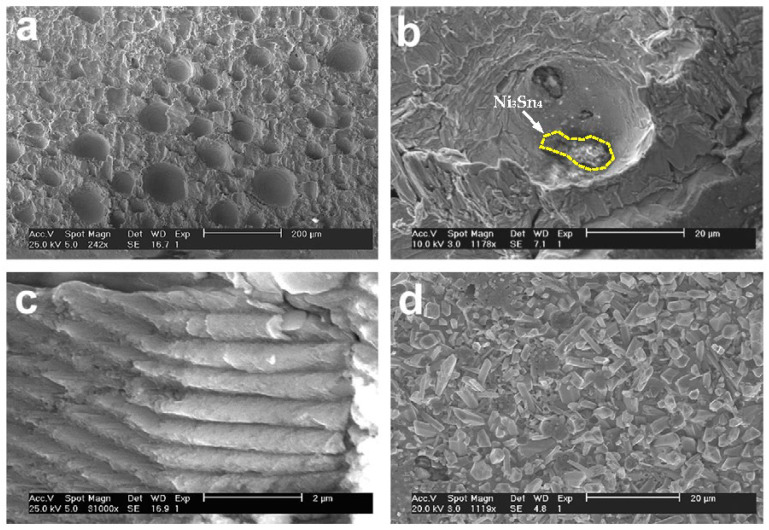
SSEM of fatigue fracture at the binding interface [29]: (**a**) bumps on solder side, (**b**) dimples on substrate side, (**c**) cross striations of fatigue, and (**d**) delaminations on substrate side.

Under thermal fatigue loads and isothermal mechanical cycles, there are obvious differences in the failed mechanism of solder joints. By the isothermal mechanical shear cycle, fatigue cracks appear in solder samples (Figure 2a). However, under 1500 cycles of thermal fatigue load, there is no obvious crack due to the significant reduction of the elastic modulus, as shown in Figure 2b [27,28]. To observe the structure of the fatigue section as much as possible, SEM observations were made on the cross sections of the alloy solder SnAgCu and the Ni/Au substrate after fatigue fracture, as shown in Figure 3. According to Figure 3a–c, all of them can be regarded as ductile fracture modes, and the formation of streaks and indentations can be seen. There is Ni_3_Sn_4_ on the tip of the scallop in the interfacial metal compound, and the solder appears delaminated on the surface of the broad. In the delaminated sample, only the IMCs formed during reflow were visible on the substrate side, as shown in Figure 3d [29].

**Figure 4:**
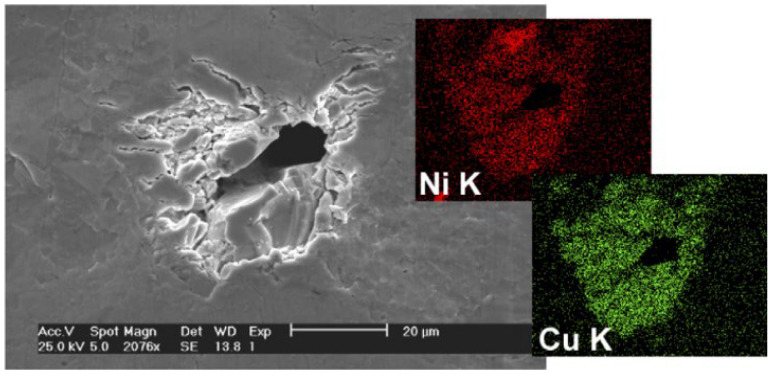
(Cu,Ni)_6_Sn_5_ IMC has broken into pieces and produced secondary cracks in the surrounding solder matrix upon tensile load [29].

For SnPb solder and substrate under the action of high–low temperature thermal cycling and shear force, the solder produces a coarse crystal zone at high temperature, and cracks are generated at the Sn-rich phase or boundary after further thermal cycling. Therefore, the high-temperature stage of thermal cycling promotes the isomerization and coarsening of the solder, resulting in cracks [30], as shown in Figure 4. Cracks spread between the interfacial metal compound layer (early stage of thermal fatigue) and the coarsely crystalline zone of the solder microstructure (after multiple thermal cycles), and its thickness has almost no effect on the thermal fatigue properties of the solder [31]. In addition, residence time at high temperatures has no significant effect on the thermal fatigue of the solder. Therefore, the accelerated thermal cycle test method can be used to study the thermal fatigue behavior of solders [32].

In microelectronic circuits, due to the different CTE coefficients of the solder and substrate that make up the electronic components, the fatigue resistance is also different. For example, the fatigue resistance of high-conductivity epoxy mold plastics filled with alumina is about six times higher than that of crystalline silicon, and the thermal expansion coefficient of polymer dielectrics and binders is larger than that of metal conductors. Significant thermo-mechanical stress is generated in through-holes or other metal interconnect structures, thus showing anisotropy, and cyclic stress is generated in the thermal cycle process, resulting in fatigue failure [33,34]. In addition, under the condition of thermal cycling, the interface strength of the FR-4 substrate coated with solder resistance is significantly reduced by thermal fatigue under the action of the shear force of the bottom filling material [35].

In addition, the method of IEC-TR-62380 reliability prediction considers the effects of phased task outlines on working and non-working components. This method also takes into account the effects of temperature cycling on the component failure degree due to changes in the environment temperature and component switch on and off. Where applicable, it also can predict the fatigue life of components.

## 3. Microstructure Evolution of Solder Joint Thermal Fatigue

A standard microelectronic production with solder joints includes a solder, a bonding pad, and an interfacial IMC layer between them. From the perspective of brazing metallurgy, it is the interfacial IMC layer formed between the solder and the broad to realize the connection of the brazing head. When the molten Sn-based solder is in contact with the pad matrix, a layer of intermetallic compound (IMC) will be formed at the interface, which is not only controlled by temperature and time in the welding process, but also its thickness will increase with the progression of time in the later service [36]. Studies have shown that fine intermetallic compounds distributed in mass in solder can improve the creep and fatigue resistance of the solder, while thick intermetallic compounds distributed in the interfacial layer are brittle, which will reduce the mechanical integrity of the interface, weaken the interface, and cause damage of solder joints at the boundary between the compound and solder, resulting in the decline of welding fracture toughness and low-cycle fatigue resistance. As a result, the reliability of solder joints decreases [37]. In this paper, the effects of thermal cycling on the thermal fatigue microstructure of solder joints are reviewed.

**Figure 5:**
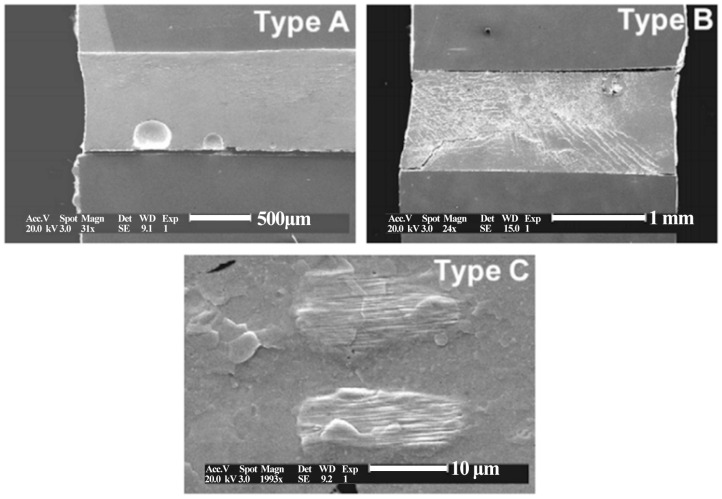
Stress decline mechanisms observed in cycling strain controlled tests and typical microstructures corresponding to A, B, and C failure types [29].

**Figure 6:**
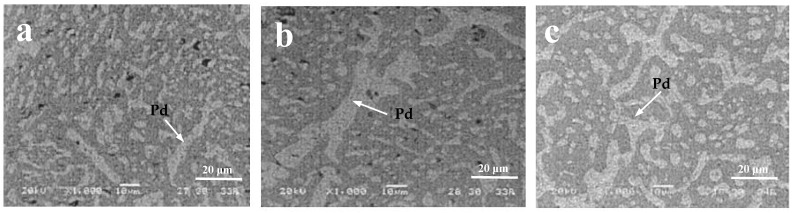
SEM of solder joint interface after different thermal cycles: (**a**) 500, (**b**) 1000, and (**c**) 2000 [39].

**Figure 7:**
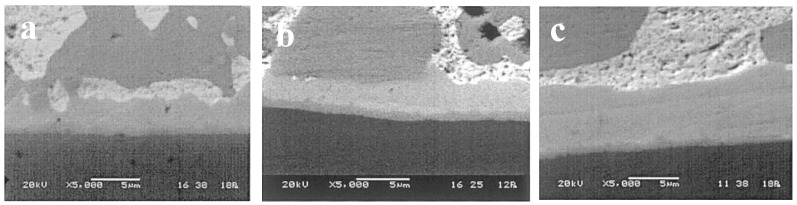
Intermetallic layers (Cu_3_Sn and Cu_6_Sn_5_) observed after different thermal cycles: (**a**) 500, (**b**) 1000, and (**c**) 2000 [39].

**Figure 8:**
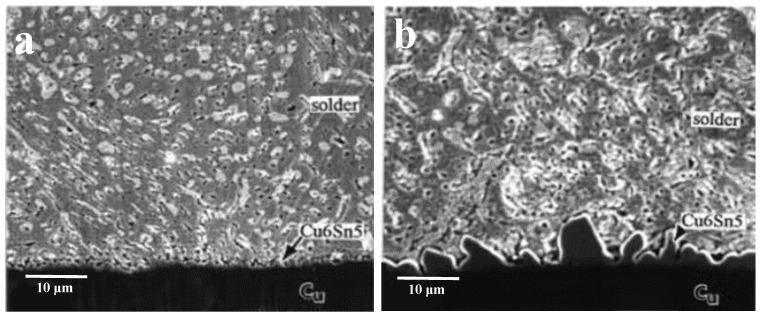
SEM of IMC interface layers of Cu-Sn solder joint after: (**a**) reflowing for 20 s and (**b**) reflowing for 600 s [40].

**Figure 9:**
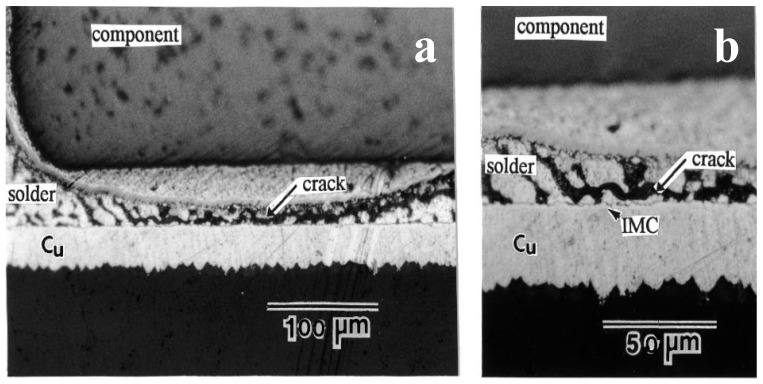
Micrographic section of the bottom fracture surface [40].

Microstructural observations suggest that there are two different failure mechanisms in operation: (a) heterogeneous matrix deformation and (b) localization of deformation at the bonding interface [38]. Failure types A, B, and C and their corresponding micrographs are shown in Figure 5 [5]. After a certain thermal cycle of SnPb solder, both the Pb phase and Sn-rich phase will undergo isomerization, and the isomerization zone can occur in any region of the interface and solder center [39], as shown in Figure 6. In addition, the coarsening behavior of the solder microstructure and the intermetallic compound layer is gradually obvious and thickened with the increase of the number of thermal cycles, as shown in Figure 7 [39]. In the process of thermal cycling, the Cu-Sn intermetallic compound layer also thickens with the increase in the number of cycles, as shown in Figure 8 [40]. Moreover, crack initiation and expansion along the interface of the IMC/solder eventually lead to the failure of solder joints, as shown in Figure 9 [40].

**Figure 10:**
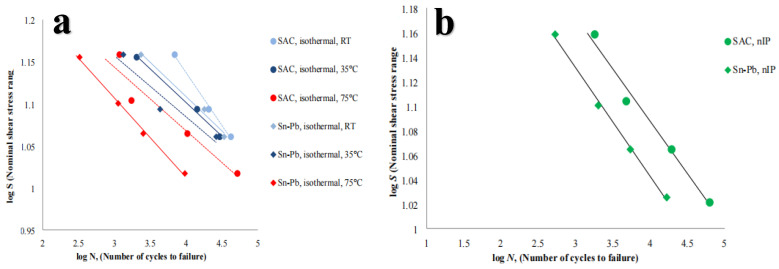
Stress–fatigue life of Sn–3.8Ag–0.7Cu (SAC) and Sn–37Pb (Sn–Pb) [44]: (**a**) under isothermal mechanical fatigue load at room temperature (RT), 35 °C, and 75 °C and (**b**) under thermo-mechanical fatigue load.

Compared with lead-free solders under thermal cycle test conditions, lead-containing solders showed a faster fatigue crack growth rate than lead-free solders, and the cracks mainly expanded in a transcrystalline manner [41,42]. However, for Sn-based lead-free solder alloy Sn-3.5Ag-X (X = Bi, Cu), low-cycle fatigue behavior was adopted. It was found that Sn-3.5Ag, Sn-0.7Cu eutectic, and Sn-3.5Ag-Cu ternary eutectic alloys showed similar fatigue resistance, and the tensile strength of Sn-3.5Ag-Cu decreased slightly with the increase of the number of thermal cycles [43]. It was also found that the alloy containing Bi solder showed poor fatigue resistance. In addition, the increase of Bi significantly reduces the fatigue resistance of Sn-3.5Ag. In addition, the Bi-containing solder alloy with the formation of Ni_3_Sn_4_ generates highly concentrated stress between layers, and its fatigue cracks extend along the IMC (Ni_3_Sn_4_/Ni_3_P) interface. As shown in Figure 10 [44], it can be observed that all the profiles show the widely expected value of life reduction as the stress scope increases. In addition, the fatigue life of both solder alloys decreases with the increase of temperature. In addition, lead-free alloys consistently have a longer fatigue life than lead-based welding alloys, and this discrepancy rises with increasing temperature. Finally, the difference in assembly performance between Pd and Pd-free tends to decrease, except at the maximum temperature, where it remains roughly the same over the study scope. Due to the temperature and mechanical cycles neither being in phase nor out of phase, the sample may be isothermally tested at an average temperature of 55 °C without additional effects.

## 4. Discussion

The reliability of solder joints under the condition of thermal cycling can be achieved through reliability tests and analyses, whose purpose is to assess and recognize the reliability capability of integrated circuit productions and offer reference data for the reliability design of the complete machine [45]. Under the condition of thermal cycling loading, due to the temperature range, frequency of thermal cycling, and the mismatch of thermal expansion coefficients between different solders and substrates, thermal stress and strain are produced inside the solder joints, resulting in thermal failure of the solder joints, thus affecting the fatigue life of the solder joints, which is a wide concern of people. In this paper, the influence factors of the thermal fatigue life of solder joints are analyzed.

**Figure 11:**
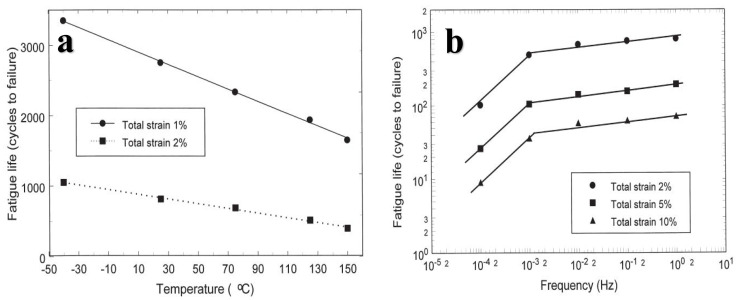
(**a**) Fatigue life versus temperature for two strain levels at 1Hz and (**b**) relation between fatigue life and frequency at a temperature of 25 °C [46].

In the process of low-cycle isothermal mechanical fatigue systematically within a certain frequency and temperature range, the low-cycle fatigue life of eutectic tin–lead solder significantly changes with the test temperature and frequency. When the temperature increases, the fatigue life linearly decreases, as shown in Figure 11a, and when the frequency is further increased from 10^−4^ Hz to 10^−3^ Hz, the fatigue life of eutectic tin–lead solder decreases linearly. The fatigue life of solder joints is significantly improved, but when the frequency is increased from 10^−3^ Hz to 1 Hz, the increase in fatigue life is small, as shown in Figure 11b [46,47].

**Figure 12:**
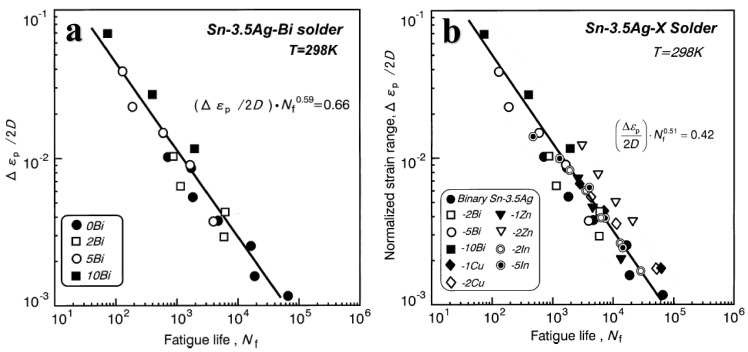
Plasticity−normalized plastic strain scope, Δε_p_/2D, as an equation of fatigue life, N_f_, for Bi-containing solders: (**a**) Sn−3.5Ag−Bi [48] and (**b**) Sn−3.5Ag−X [49].

When Bi, Cu, Zn, and In alloy elements are added to the Sn-3.5Ag eutectic solder, the plasticity of the alloy decreases obviously with the addition of Bi content, while the tensile strength enhances greatly. However, the fatigue life of the solder depends on the ductility obtained through the tensile test, so the fatigue life of the solder decreases significantly with the increase of Bi content [48], as shown in Figure 12a. In addition, the effects of Cu, Zn, and In contents on the fatigue life of Sn-3.5Ag alloys have been found. The increase of Zn and In content also reduces the fatigue life of the alloy due to the loss of ductility, as shown in Figure 12b [49]. The fatigue life can be predicted by the Coffin–Manson empirical equation [50]. The Coffin–Manson equation is an empirical formula used to predict the life of a material under fatigue loading, and its expression is shown in (1).

Δε/2 = C(N_f_)k (1)

In the formula, Δε/2 represents half of the strain amplitude, N_f_ represents the number of stress cycles, C is a constant related to the material properties, and k is a constant representing the brittleness of the material. The basic idea of this formula is that the damage of the material under fatigue loading is mainly caused by plastic deformation, when the material receives stress loading, a series of stress cycles will occur, and each cycle will cause a certain degree of plastic deformation. These plastic deformations will cause the accumulation of microscopic damage and defects inside the material and eventually lead to fatigue damage [51].

## 5. Simulation Analysis and Prediction of Fatigue Life of Solder Joints

Fatigue life prediction has always been regarded as an important content of chip package reliability research. In the process or service process of electronic packaging and its components, the thermal expansion mismatch of materials will produce thermal stress/strain in the package structure, resulting in electrical, thermal, and mechanical failure of electronic packaging, so the reliability of electronic packaging is one of the key issues in this technical field [26,52]. The fatigue life of electronic packaging products is the key to determining its reliability, and the fatigue life can be determined through theoretical analysis and numerical simulation. The theoretical analysis methods mainly include the Green function method, the separate variable method, and the semi-analytical method, and the numerical simulation is mainly developed from the iterative numerical technology to the present finite element method or finite difference method. The reliability data of new products can be obtained through finite element simulations, so there is no need to perform reliability experiments after making actual samples, thus saving a lot of development costs and shortening the product development period [53,54]. In this paper, the author makes a life prediction based on the simulation analysis of the thermal fatigue of solder joints. Numeric analysis is performed with 1% alternating cyclic strain for 5000 cycles. An effective injury value *D*_eff_ at each adhesive zone is calculated from the direct and circumferential damage samples according to the following relation (2):

(2) $$D_{\mathrm{eff}}={\left(D_{t}^{2}+D_{n}^{2}-D_{t}D_{n}\right)}^{\frac{1}{2}}$$

**Figure 13:**
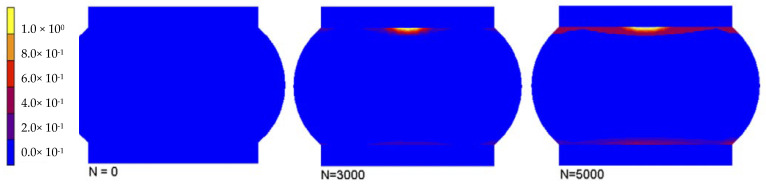
Effective damage (*D*_eff_) of cyclic shear strain in a SnAgCu solder after cycles of 0, 3000, and 5000 [29].

The contour graphs of effective damage *D*_eff_ are shown in Figure 13, when the number of cycles N are 0, 3000, and 5000. The values of the visibility node are linearly equated in the contour graph. Therefore, the damage is preformed visible by the adjacent contiguous elements, only for visualization aims.

For the prediction of the solder fatigue life, researchers use various test methods (such as the combination of mechanical vibration and thermal cycling load test, fracture mechanics and disturbed state mixed method, and global and local methods) to investigate the deformation of solder joints under cyclic load and develop special test equipment for reliability assessment to predict the solder fatigue life [55,56,57,58]. Another researcher designed a hybrid fatigue modeling method to simulate the crack trajectory of solder joints and predict its fatigue life, which has been verified by lead-free and tin–lead solders and has achieved good results [59,60].

It is worth pointing out that many researchers have substituted the unified constitutive model of solder joints for mechanical reliability tests and used finite element programs for predictive analysis, and combined with actual experimental data, the fatigue life of solders can be predicted considering various load factors [61,62,63,64].

There are several packaging designs and establishments that can be obtained from the current research to ultimately enhance its reliability. Additive layers can be injected with different material choices that will obviously contribute to the decrease of stress on the solder. Examples of improved design are the combination of innovative technologies. For example, making a notch on one side of the copper column and changing the height of the post and stud. These educed designs and structures can be investigated by finite element analysis (FEA) to study their effects on the reliability of solder joints.

Since the broadening of applications of new semiconductor packaging devices, analytical methods of fatigue life can also be deepened such as in space technology applications, repeated drop tests, repetitive bending tests, and cyclic moisture exposure tests.

There are existing fatigue models obtainable for the fatigue analysis. The fatigue models and their distinctions are summarized in Table 2. These models can be further exploited by the incorporation of three or more equations to realize a mixed fatigue model that can be applied for CSP, BGA or IGBT.

## 6. Conclusions and Prospect

With the increasing use of microelectronic devices today, the reliability of packaging is a key issue that must be considered, and the thermal fatigue research of microelectronic solder joints has been become a hot issue. It has been shown that under the action of alternative temperature thermal cycles and shear force, the fatigue resistance of solder and substrate is different due to the different CTEs. Under thermal fatigue load and isothermal mechanical cycling, there are obvious differences in the failure mechanisms of solder joints. In the high-temperature stage, the isomeric coarsening of the solder is promoted, resulting in fatigue cracks, and the cracks expand in the interlayer metal compound and the coarse crystal interval. However, the thickness of the solder and its residence time in the high-temperature stage have no significant influence on thermal fatigue. Therefore, the thermal fatigue behavior of the solder can be studied using accelerated thermal cycle tests. After thermal cycling, the solder will be isomerized and coarsened between metal compound layers, and the coarse crystal zone and IMC layer formed will be obviously thickened with the increase of cycle times. Cracks will start and expand along the solder/IMC interface, and eventually lead to solder joint failure. For leaded and lead-free solders, fatigue cracks with lead showed a faster growth rate and spread in a transgranular manner. Temperature and frequency have different degrees of influence on the fatigue life of the solder joint; when the temperature increases, the life decreases, and the frequency has a significant effect on the low value, but the influence is not obvious when reaching a certain value. In addition, other alloying elements also have an effect on the fatigue life of the solder joint, such as Bi, Cu, Zn, and In, and the fatigue life of the solder joint will be decreased with their increased content. The fatigue life of solder joints can be predicted by testing the deformation of solder joints by various methods and simulating crack trajectories. The fatigue life of solder joints can also be predicted by establishing a unified model and using FEA combined with the Coffin–Manson empirical formula. On the one hand, the in-depth study of the above problems helps to reveal the failure mechanism and influence factors of thermal fatigue of solder joints and explain the effect on the microstructure of thermal fatigue failure. On the other hand, predicting the reliability and fatigue life of solder joints is significant.

## Figures and Tables

**Table 1:** Mechanical properties of SAC based on different additives used [5].

SAC Alloys	Additives	Percent Composition	Shear Stress	Tensile Stress	Hardness	Creep Resistance	Reference
Sn0.3Ag0.7Cu	Al_2_O_3_ nano	0.01–0.5	increase	increase			[15]
Sn0.5Ag0.7Cu	Ga	0.05–1.0	increase				[16]
Sn0.5Ag0.7Cu0.5Ga	Pr	0.06–0.5	increase				[17]
Sn0.7Ag0.5Cu	Bi and Ni	3.5, 0.05				increase significantly	[18]
Sn1.0Ag0.3Cu	Fe	0.1–0.5		decrease significantly			[19]
Sn1.7Ag0.7Cu	Co	0.5		increase			[20]
Sn3.0Ag0.5Cu	SnO_2_ nano	0.1–1.0			increase significantly	increase significantly	[21]
Sn3.0Ag0.7Cu	Fe, Te, Co, and B	0.1, 0.2				increase significantly	[22]
Sn3.5Ag0.5Cu	ZnO nano	0.5		increase			[23]
Sn3.5Ag0.7Cu	Ga	1.5	increase				[24]
Sn3.8Ag0.7Cu	Al nano	0.1–0.4	increase	increase		increase significantly	[25]

**Table 2:** Distinctions of different fatigue models [5].

Fatigue Model	Strain		Energy	Damage
	Plastic	Creep		
Coffin–Manson	●			
Total strain	●			
Soloman	●			
Engelmaier	●			
Miner	●	●		
Knecht and Fox		●		
Syed		●	●	
Akay			●	
Liang			●	
Heinrich			●	
Pan			●	
Darveaux			●	●
Stolkarts				●

●: The mean is corresponding to fatigue model.

## Data Availability

Not applicable.
